# High pre-diagnosis attrition among patients with presumptive MDR-TB: an operational research from Bhopal district, India

**DOI:** 10.1186/s12913-017-2191-6

**Published:** 2017-04-04

**Authors:** Deepak Shewade, Arun M Kokane, Akash Ranjan Singh, Manoj Verma, Malik Parmar, Ashish Chauhan, Sanjay Singh Chahar, Manoj Tiwari, Sheeba Naz Khan, Vivek Gupta, Jaya Prasad Tripathy, Mukesh Nagar, Sanjai Kumar Singh, Pradeep Kumar Mehra, Ajay MV Kumar

**Affiliations:** 1 International Union Against Tuberculosis and Lung Disease (The Union), South-East Asia Office, New Delhi, India 110016; 2 Department of Community Medicine and Family Medicine, All India Institute of Medical Sciences (AIIMS), Bhopal, India; 3 State TB cell, Department of Health and Family Welfare, Bhopal, India; 4 World Health Organization, Country Office in India, New Delhi, India; 5 Department of Community Ophthalmology, All India Institute of Medical Sciences (AIIMS), New Delhi, India; 6 International Union Against Tuberculosis and Lung Disease, Paris, France

**Keywords:** Tuberculosis, multidrug-resistant/diagnosis, Tuberculosis, multidrug-resistant/prevention and control, Diagnosis, delayed, Operational research, India, Attrition, Diagnosis and treatment pathway

## Abstract

**Background:**

Pre-diagnosis attrition needs to be addressed urgently if we are to make progress in improving MDR-TB case detection and achieve universal access to MDR-TB care. We report the pre-diagnosis attrition, along with factors associated, and turnaround times related to the diagnostic pathway among patient with presumptive MDR-TB in Bhopal district, central India (2014).

**Methods:**

Study was conducted under the Revised National Tuberculosis Control Programme setting. It was a retrospective cohort study involving record review of all registered TB cases in Bhopal district that met the presumptive MDR-TB criteria (eligible for DST) in 2014. In quarter 1, Line Probe Assay (LPA) was used if sample was smear/culture positive. Quarter 2 onwards, LPA and Cartridge-based Nucleic Acid Amplification Test (CbNAAT) was used for smear positive and smear negative samples respectively. Pre-diagnosis attrition was defined as failure to undergo DST among patients with presumptive MDR-TB (as defined by the programme).

**Results:**

Of 770 patients eligible for DST, 311 underwent DST and 20 patients were diagnosed as having MDR-TB. Pre-diagnosis attrition was 60% (459/770). Among those with pre-diagnosis attrition, 91% (417/459) were not identified as ‘presumptive MDR-TB’ by the programme. TAT [median (IQR)] to undergo DST after eligibility was 4 (0, 10) days. Attrition was more than 40% across all subgroups. Age more than 64 years; those from a medical college; those eligible in quarter 1; patients with presumptive criteria ‘previously treated – recurrent TB’, ‘treatment after loss-to-follow-up’ and ‘previously treated-others’; and patients with extra-pulmonary TB were independent risk factors for not undergoing DST.

**Conclusion:**

High pre-diagnosis attrition was contributed by failure to identify and refer patients. Attrition reduced modestly with time and one factor that might have contributed to this was introduction of CbNAAT in quarter 2 of 2014. General health system strengthening which includes improvement in identification/referral and patient tracking with focus on those with higher risk for not undergoing DST is urgently required.

**Electronic supplementary material:**

The online version of this article (doi:10.1186/s12913-017-2191-6) contains supplementary material, which is available to authorized users.

## Background

Globally, tuberculosis is a major public health problem and the emergence of multidrug-resistant/Rifampicin resistant tuberculosis (MDR/RR-TB) poses a major threat to the control of TB. Access to Drug Susceptibility Testing (DST) for patients with TB has increased in recent times. Despite this, gaps remain in the diagnosis and treatment pathway (DTP) of MDR-TB. In 2015, of the estimated 580,000 MDR/RR-TB among notified TB cases globally, 132,120 (23%) were diagnosed and of them, 125,000 (95%) were initiated on treatment [1]. This indicates a huge gap in diagnosis of MDR-TB: India, Indonesia and Nigeria alone accounting for almost half of the gap. Studies worldwide have raised concerns over high attrition and/or delays in MDR-TB DTP [2–8].

India has the highest burden of TB as well as MDR-TB and accounts for more than one fourth of the global burden [1]. The Revised National Tuberculosis Control Programme (RNTCP) has adopted the World Health Organization (WHO) recommended Programmatic Management of Drug-resistant TB (PMDT) for effective delivery of drug resistant tuberculosis services [9]. Prompt identification of patients with presumptive MDR-TB (one who is eligible for DST), diagnosis of MDR-TB and initiation of treatment are crucial to prevent the transmission of disease and reduce high morbidity and mortality [10]. There were an estimated 130,000 MDR-TB cases in 2015. Only 28,876 cases were detected giving a case detection rate of 22% and a total of 24,396 (84%) were put on treatment [1, 11]. If TB management practices across sectors remain unchanged for the next 20 years in India, it has been predicted that there will be an increase in MDR-TB incidence, untreated MDR-TB prevalence and risk of MDR-TB by 152, 242 and 275% respectively [12].

RNTCP has limited cohort-wise information about what happens to the patients with presumptive MDR-TB. There is also paucity of data regarding the delays and factors causing attrition in the process. To our knowledge, there are only three published studies on this issue from India [3, 13, 14]. Operational challenges are unique and differ from region to region especially in a large country like India. Addressing them will aid program managers working at national and local level to strengthen PMDT services.

Here we report the findings related to diagnostic pathway of DTP among patients with presumptive MDR-TB in Bhopal District, central India for the year 2014. Findings related to pre-treatment attrition will be reported in a separate paper. Specific objectives were to determine the i) number (proportion) with pre-diagnosis attrition ii) turn-around time (TAT) for various steps in diagnosis (including time to get DST) and iii) clinical and demographic factors associated with pre-diagnosis attrition.

## Methods

### Study Setting

#### General Setting

Bhopal district is situated in the state of Madhya Pradesh, the second largest state in India. Bhopal district with population of 2.53 million is predominantly urban. RNTCP infrastructure includes one District TB Center (DTC), five sub-district level programme management units (Tuberculosis Units - TU) and 24 designated microscopic centers (DMCs) for sputum acid fast bacilli examination. Among 24 DMCs, six are located in medical colleges, five in district level hospital and 13 in primary/secondary level health centers. Once a patient is diagnosed with TB, a TB-Health Visitor from the DMC ensures initiation of treatment after address verification followed by regular monitoring.

#### PMDT services

In Bhopal district (2014), the diagnostic facility (National Reference Laboratory - NRL) is located in a tertiary, public health care facility, named Bhopal Memorial Hospital and Research Center. The diagnostic facility is accredited by the RNTCP for phenotypic (solid/liquid culture and DST) and molecular diagnostic techniques (Line Probe Assay – LPA and Cartridge-based Nucleic Acid Amplification Test - CbNAAT). CbNAAT was introduced from quarter 2 of 2014. In quarter 1, if sample was smear positive, then LPA was used upfront. Among smear negative samples, culture was done followed by LPA, if culture turned out to be positive. Quarter 2 onwards, LPA was used for smear positive and CbNAAT was used for smear negative samples.

Treatment for MDR-TB was provided at DR-TB center at TB hospital, Bhopal according to RNTCP PMDT guidelines which were in the line with WHO recommendations [10]. Patients with RR-TB were also treated with the standard MDR-TB regimen. Therefore, in the study MDR-TB included RR-TB as well.

Patients with presumptive MDR-TB included all ‘previously treated’ patients, any patient who was follow-up smear positive (FUS+), new patients with pulmonary TB who were contacts of known MDR-TB and all HIV-TB co-infected cases at diagnosis (criterion C as per PMDT 2012 guidelines). Criterion C was implemented from the second quarter of 2014 in Bhopal. HIV-TB co-infected cases and smear negative ‘previously treated’ patients were excluded from the presumptive MDR-TB criteria in quarter one of 2014. (criterion B).

All DMCs served as sputum collection centers for DST. Once identified and referred as presumptive MDR-TB at DMC, the TB-Health Visitor has been assigned the responsibility of getting the patient’s sample transported to NRL along with a request for culture and DST form, a copy of which is maintained at the DMC. DST for ‘previously treated’ patients was done before being registered for TB treatment: based on DST results, patients were either registered on TB or DR-TB treatment [10]. DR-TB supervisor at district level maintained a line list in ‘referral for DST’ register and ensured treatment initiation of diagnosed MDR-TB patients.

Case definitions used in this study have been described in Table 1.

**Table 1:** Case definitions used in this study, Bhopal, India (2014) [19]

New case – A patient with TB who has never had treatment for TB or has taken anti-TB drugs for less than one month
Previously treated patients (received one month or more of anti-TB drugs in the past)
Recurrent TB – A patient with TB previously treated and declared as successfully treated (cured/treatment completed) and is subsequently found to be microbiologically confirmed TB case
Treatment after failure – Previously treated and whose treatment failed at the end of their most recent course of treatment
Treatment after loss to follow up – A previously treated patient and was declared loss to follow up in their most recent course of treatment and subsequently found microbiologically confirmed TB case
Others – A previously treated patient with TB but whose outcome after their most recent course of treatment is unknown or undocumented. (This subgroup mostly refers to previously treated patients who are smear negative)
Follow-up smear-positives (FUS+) – A patient with TB whose follow up sputum is positive during any of the routine follow up
HIV associated with TB/HIV-TB co-infected cases – A patient with TB who is a previous known case of HIV or gets diagnosed as HIV during diagnosis of TB or anytime during TB treatment

### Study design and study population

It was a retrospective cohort study involving record review of all patients with TB registered for treatment under RNTCP and met the presumptive MDR-TB criteria (DST-eligible patients) between 1 January 2014 and 31 December 2014 at Bhopal district.

### Data variables, sources of data and data collection

Data were collected during September 2015-March 2016. At TU level, a list of eligible patients was prepared by investigators based on the information from the TB treatment register (at TU). For the presumptive MDR-TB criterion ‘new patients with pulmonary TB who were contacts of known MDR-TB’, we referred to the list maintained by the DR-TB supervisor and details recorded in the DR-TB treatment cards.

Each eligible patient in the list was tracked in the records at DTC (referral for DST register), NRL (Culture and DST register) and DR-TB center (PMDT treatment register). Data for each eligible patient was consistently reviewed for three months post the date of eligibility. Review period was extended to additional three months in case of invalid result or smear negative sample.

The variables, corresponding sources of data and operational definitions have been summarized in Table 2.

**Table 2:** Source of data collection and operational definition of variables collected for patients with presumptive/confirmed MDR-TB, Bhopal district, India (2014)

Variables	Source	Operational definition
Date of eligibility for DST, presumptive MDR-TB patient criteria, age in completed years, sex, TB registration number, year of registration, DMC name, baseline smear status	Treatment register	Under ‘previously treated’ criterion, for smear positive patient, date of smear examination was the date of eligibility. For smear negative patient, date of treatment initiation was the date of eligibility. Under TB/HIV, those with HIV first and TB later, date of eligibility depended on whether the patient was smear positive or negative and we followed the above mentioned definition. For those with TB first and then HIV, date of HIV testing was considered. For patients with known MDR-TB contacts, date of TB registration was considered. Follow up smear positive at 5 months was considered as ‘previously treated’ and included as eligible patient if the date of eligibility under ‘previously treated’ criterion was in 2014.
Whether referred for DST, date of referral for DST	Referral for DST register (DTC) or copy of request for DST form (DMC)	If there was a record for referral maintained at DTC or DMC then it was considered as ‘identified/referred’. In case of discrepancy in dates, earlier date was considered.
Sputum received at NRL, date of sputum received at NRL, whether DST was performed, type of DST, date of DST, DST result, date of DST result, date of dispatch of DST result to DTC	DST register at NRL	Eligible patients with presumptive MDR-TB were tracked through their TB registration numbers; in cases where it was not entered, name and address of the patient was used. If NRL DST register showed ‘contaminated’ as the result and no further sample was received then it was recorded as ‘sample received; DST not done’.
Whether patient referred to DRTB center from DTC. date of referral to DRTB center	Referral for DST register (DTC)	-
Whether treatment initiated, DR-TB treatment. date of treatment initiation	Treatment register at DRTB center	-

*MDR-TB* Multi drug-resistant tuberculosis, *DMC* Designated microscopy center, *DST* Drug susceptibility testing, *NRL* National reference laboratory, *DTC* District tuberculosis center, *DRTB* Drug-resistant tuberculosis

### Data management and statistical analysis

Real time data capture was enabled through data entry in a shared dropbox folder (http://www.dropbox.com/) [15]. The International Union Against Tuberculosis and Lung Disease (The Union), South-East Asia Office, coordinated this process with the investigators from Bhopal.

Data collected (provided as Additional files 1 and 2) in a pre-tested, structured form were double entered, validated and analyzed using EpiData (version 3.1 for entry and version 2.2.2.183 for analysis, EpiData Association, Odense, Denmark) for descriptive and unadjusted analysis. Multivariable adjusted analysis was done using STATA (version 12.1 STATA Corp., College Station, TX, USA).

**Fig. 1:**
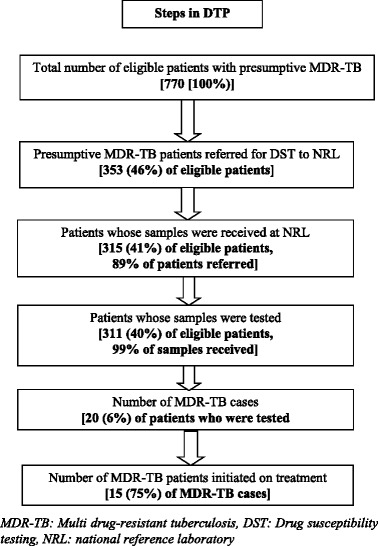
Flow of presumptive/confirmed MDR-TB patients in the diagnosis and treatment pathway, District Bhopal, India (2014)

Pre-diagnosis attrition was defined as patients with TB at risk for MDR-TB (as per the programme’s own definition) who failed to successfully have a DST conducted. Key analytic outputs were number (proportion) of eligible patients at each step of DTP (Fig. 1); median (IQR) Turnaround Time (TAT) in days for each step; and association between not getting tested and various clinical and demographic factors. Adjusted analysis was done by fitting the variables in poisson regression with robust variance estimates (enter method). Unadjusted and adjusted Relative Risks (RRs) were reported with 95% confidence intervals (CI).

## Results

There were 770 patients eligible for DST: mean age (SD) in years was 37 (15) and 520 (68%) were males. Criteria for eligibility for DST was ‘previously treated TB’ in 635 (83%) cases. These patients were from DMCs located in primary/secondary level health facility in 362 (47%) cases and district level facility in 273 (36%) cases (Table 3)

**Table 3:** Clinical and demographic profile of patients with presumptive MDR-TB, District Bhopal, India (2014)^a^

Variable	Patients with presumptive MDR-TB	
	Number	Percentage
Total	770	100
Age (years)		
• <14	7	1
• 14-44	513	67
• 45-64	203	27
• ≥65	47	6
Gender		
• Male	520	68
• Female	250	33
Health facility		
• Primary/Secondary level	362	47
• District level	273	36
• Medical college	135	18
Presumptive MDR-TB criteria		
• Previously treated TB	635	83
○ Recurrent	251	33
○ Treatment after Loss to follow up	51	7
○ Treatment after Failure	22	3
○ Others	311	40
• Follow up smear positives	132	17
• New patient with TB/HIV	3	0
• New pulmonary TB withknown MDR-TB contact	0	0
Site of Tuberculosis		
• Extra pulmonary	89	12
Pulmonary – smear negative	248	32
• Pulmonary – smear positive	433	56
Quarter		
• January – March 2014	101	13
• April – June 2014^b^	238	31
• July – September 2014	223	29
• October – December 2014	208	27

^a^ *MDR-TB* Multi drug-resistant tuberculosis, *TB* Tuberculosis, *HIV* Human Immunodeficiency Virus ^b^LPA was used as DST for smear positive patient; Cb-NAAT was introduced as DST for smear negative patient in quarter 2

**Fig. 2:**
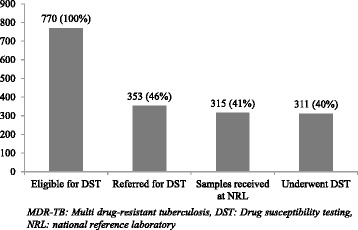
Cascade of care of patients with presumptive MDR-TB (eligible for DST) in the diagnosis pathway, District Bhopal, India (2014)

Of the eligible, 46% (353/770) were identified/referred by the programme. Of the referred, 11% (38/353) samples did not reach the NRL. Of the samples received (*n* = 315), 311 (99%) got tested. Pre-diagnosis attrition was 60% (459/770) among eligible. Failure to identify eligible patients contributed to 91% (417/459) of the pre-diagnosis attrition. There were 20 MDR-TB patients diagnosed. Of them, pre-treatment attrition was seen in five (25%) (Figs. 1 and 2).

TAT for specific steps between eligibility and testing have been described in Table 4. TAT [median (IQR)] to DST from date of eligibility was 4 (0, 10) days.

**Table 4:** Turnaround time for various steps in diagnosis pathway of patients with presumptive MDR-TB, District Bhopal, India (2014)

Variable	Number of	Days
	Patients^a^	Median (IQR)
Patients with presumptive MDR-TB	770	-
Days to refer from date of eligibility	353	2 (0,8)
Days to receive sputum at NRL from referral	315	0 (0,0)
Days to test at NRL from sputum receipt	310	0 (0,2)
Days to dispatch result from NRL from testing	304	2 (0,4)
Days to test at NRL from date of eligibility	310	4 (0,10)

*MDR-TB* Multi drug-resistant tuberculosis, *DMC* Designated microscopy centre, *NRL* National reference laboratory

^a^Includes patients who completed the respective process and whose respective dates were recorded

On unadjusted analysis, factors associated with not getting tested were: elderly patients (≥65 years of age); patients with criterion ‘previously treated – loss to follow up’ and ‘previously treated – others’; and patients with extra pulmonary and smear negative pulmonary TB (Table 5).

**Table 5:** Clinical and socio-demographic factors associated with not getting DST among patients with presumptive MDR-TB, District Bhopal, India (2014)

Variable	Total	Not tested for	RR (0.95 CI)	aRR (0.95 CI)^a^
	[n]	DST [n (%)]		
Total	770	459 (60)		
Age (years)				
• <14	7	4 (57)	0.9 (0.5, 1.9)	1.0 (0.6, 1.9)
• 14-44	513	301 (59)	1.0 (0.9, 1.2)	1.0 (0.9, 1.2)
• 45-64	203	118 (58)	Ref	Ref
• >/= 65	47	36 (77)	1.3 (1.1, 1.6)*	1.3 (1.1, 1.7)*
Gender				
• Male	520	314 (60)	1.0 (0.9, 1.2)	1.1 (1.0, 1.2)
• Female	250	145 (58)	Ref	Ref
Health facility				
• Primary/Secondary level	362	208 (58)	1.0 (0.9, 1.1)	1.0 (0.9, 1.1)
• District level	273	159 (58)	Ref	Ref
• Medical college	135	92 (68)	1.2 (1.00, 1.4)	1.2 (1.02, 1.4)*
Presumptive MDR-TB criteria				
• Previously treated – recurrent	251	128 (51)	1.2 (0.9,1.5)	1.3 (1.0, 1.6)*
• Treatment after failure	22	11 (50)	1.2 (0.7, 1.9)	1.3 (0.8, 2.0)
• Treatment after loss to follow up	51	33 (65)	1.5 (1.2, 2.0)*	1.5 (1.1, 2.1)*
• Previously treated – others	311	229 (74)	1.7 (1.4, 2.1)*	1.6 (1.1, 2.3)*
• Follow up smear +	132	56 (42)	Ref	Ref
• New patient with TB-HIV	3	2 (67)	1.6 (0.7,3.6)	1.8 (0.8, 4.2)
Site of Tuberculosis				
• Extra pulmonary	89	72 (81)	1.6 (1.4, 1.9)*	1.5 (1.0, 2.2)*
• Pulmonary – smear negative	248	172 (69)	1.4 (1.2, 1.6)*	1.2 (0.8, 1.7)
• Pulmonary – smear positive	433	215 (50)	Ref	Ref
Quarter				
• January – March 2014	101	63 (62)	Ref	Ref
• April – June 2014^b^	238	144 (61)	0.97 (0.8, 1.2)	0.8 (0.7, 0.98)*
• July – September 2014	223	132 (59)	0.95 (0.8, 1.1)	0.8 (0.7, 0.99)*
• October – December 2014	208	120 (58)	0.9 (0.8, 1.1)	0.8 (0.6, 0.95)*

*MDR-TB* Multi drug-resistant tuberculosis, *DST* Drug susceptibility testing

^a^adjusted relative risk calculated using poisson regression with robust variance estimates (enter method)

*p < 0.05

^b^LPA was used as DST for smear positive patient; Cb-NAAT was introduced as DST for smear negative patient in quarter 2

On adjusted analysis, elderly patients eligible for DST were at 30% higher risk for not getting tested when compared to patients of 45–64 age groups. When compared to patients referred from a district-level facility, those referred from a medical college had 20% higher risk of not getting tested. When compared to patients with presumptive criterion ‘FUS+’, those with criterion ‘previously treated – recurrent TB’, ‘previously treated – loss to follow up’ and ‘previously treated – others’ were 30, 50 and 60% more unlikely to get tested respectively. Patients with extra pulmonary TB had 50% higher risk of not getting tested when compared to patient with pulmonary TB. After first quarter of 2014, the risk of not getting tested reduced by 20% (Table 5).

## Discussion

Among patients with presumptive MDR-TB listed from records, there was high pre-diagnosis attrition; majority contributed by failure to identify/refer patients by the programme. There was a modest reduction in attrition in quarter 2 of 2014 and onwards – this might be related to introduction of Cb-NAAT. Eligible patients with ‘recurrent TB’, ‘treatment after loss to follow up’, ‘previously treated-others’ criteria and extra pulmonary TB were less likely to undergo DST. If the patient was old and/or referred from a medical college, there were higher chances of pre-diagnosis attrition.

### Strengths of the study

The study had several strengths. This was an operational research study under the programme conditions using programme staff. Methodology used was robust with pre-defined operational definitions and a clear and uniform follow-up period (3 months) defined for record review. We began with identification of patients who were eligible for DST and then looked at how many of those were identified/referred by the programme. If we would have gone by eligible patients referred for DST as the denominator then the pre-diagnosis attrition would have been 12% (42/353): which does not reflect the true picture. This aspect has been missed by most of the studies on this issue [2, 3, 5, 6]. Data was quality assured and robust as double data entry and validation was done. Since we studied the entire population of patients with presumptive MDR-TB available from records in Bhopal without any sampling, the results are likely to be representative and reflect the ground reality for the region and have implication for policy. STROBE guidelines were followed for the conduct and reporting of this OR [16].

### Limitations of the study

Among patients eligible for DST, only three patients had presumptive criteria ‘new patient with TB/HIV’. However, this correlated with the information in the annual performance report for Bhopal in 2013 (HIV prevalence was 0% among notified patients with TB) [17]. There were no patients under the criterion “patients with close contact of known MDR-TB”. Currently, this information is not systematically captured in any of the programme records. Since our study relied on record reviews, it was challenging to obtain this information. As we prepared the list of patients eligible for DST from TB treatment registers in TU, we would have missed patients diagnosed at DMCs who did not get registered in TUs (pre-treatment loss to follow-up). A small percentage of patients might have got wrongly categorized as “new” patients, even when they actually had a prior treatment history, and our research approach was not designed to capture these patients [18]. Barriers related to access including distance of patient’s residence to DMC, NRL and travel costs were not collected as this information is not routinely collected and hence, beyond the scope of this OR. There are inherent limitations of a record review study, but records in RNTCP are monitored and supervised which includes periodic data validation.

### Key findings

The main cause of attrition was the large gap in identification/referral of eligible patients by the programme. Minimal attrition at the level of NRL and low TAT for testing meant the performance of diagnostic pathway was satisfactory post referral. However, in Puducherry, India, the main cause of attrition was the gap in reaching the diagnostic facility after referral [13]. This is vital as this informs the programme regarding whether there was scope to improve the gap in identification or the gap in sample reaching the NRL after referral.

Attrition was more than 40% across all subgroups. Smear negative previously treated patients were added to the eligibility criteria in second quarter of 2014. There was a lack of clarity among the programme staff regarding the criteria for presumptive MDR-TB. This is justified by our finding that extra pulmonary TB patient and patients with criterion ‘previously treated-others’, had high risk of not getting tested. (Table 5) With regards to extra-pulmonary TB even the national PMDT guidelines were not clear at that time: what specimens should be collected and the methods for storage and processing before sending to the laboratory [10]. The recently released technical and operational guidelines (2016) have incorporated this point [19].

Among eligible patients that were referred, we found that TB registration number was not recorded in referral for DST form, referral for DST register and laboratory register at NRL. This made it difficult for the investigators to track the patients. This could be explained for ‘previously treated’ patients by the fact that the programme recommends that smear positive previously treated patient may be directly registered on MDR-TB treatment if DST results are expected within 7 days (as done in Bhopal which used LPA/CbNAAT) [10]. However, for other presumptive MDR-TB criteria, TB registration number is available at the time of referral. Unlike the finding in Puducherry, India [11], referral for DST register was maintained in Bhopal.

Twenty patients were eligible for DR-TB treatment from our presumptive MDR-TB cohort (*n* = 770). There were 74 MDR-TB diagnosed from Bhopal in 2014 (based on date of diagnosis). Some of these were not part of our cohort because the date of eligibility (for DST) of these patients was not in 2014. Many of them were not part of our cohort because they were not registered for TB treatment (directly sent for DST and registered on DR-TB treatment). Pre-diagnosis attrition in our cohort was 60% (459/770). Even on inclusion of these additional MDR-TB patients (*n* = 52) into our cohort (assuming the drug-susceptible patients among those who were directly sent for DST would have been eventually registered under previously treated TB and included in out cohort), the pre-diagnosis attrition would be 56% (459/822); not a programmatically significant difference form 60%. The estimate of pre-treatment attrition (5/20) and TAT to treat among MDR-TB (*n* = 20) from our cohort may not be representative of the true picture and we intend to conduct a separate study among all the diagnosed patients of Bhopal in 2014 (based on date of diagnosis).

There is a call for universal DST in the post-2015 End TB Strategy [20]. India should aim to make DST accessible to all patients with TB in coming few years [21]. This requires strengthening of laboratories and rapid uptake of rapid diagnostics like LPA and CbNAAT, as well as use of information and communication technology to improve completeness of reporting [20]. A study conducted by Central TB Division revealed that universal DST using CbNAAT, up front to patient with presumptive TB, increased MDR-TB case notification five folds [22]. Central TB Division also plans to implement universal DST to all presumptive MDR-TB followed by DST guided treatment in select districts with good DOTS outcomes [23]. Despite the use of molecular techniques in Bhopal, pre-diagnosis attrition was high, similar to Puducherry, India [13]. Attrition reduced modestly with time and one factor that might have contributed to this was introduction of CbNAAT (Table 5). Therefore, addressing programmatic factors to improve timely identification of eligible patients and prompt transport of quality specimen is a pre-requisite as technology alone cannot lead to universal access.

### Implementation strengthening

Prevention of emergence of MDR-TB in the community is of greater priority than its treatment and RNTCP recognizes this [10]. However, it is also important to promptly identify and treat MDR-TB early enough, which is a challenge. Pre-diagnosis attrition needs to be addressed urgently along with lab capacity/technology expansion if we are to make progress in improving MDR-TB case detection and achieve universal access to MDR-TB care [24, 25].

Keeping this in mind and our study findings, we would like to make the following recommendations: i) Health system strengthening including training and re-sensitizing the staff of general health care delivery system, especially medical officers of peripheral health institutes and laboratory technicians of DMCs so that eligible patients are identified with focus on elderly, medical colleges and extra pulmonary TB ii) strengthen mechanism of sputum transport from DMC to NRL to address the issue of attrition after referral (Figs. 1 and 2): considering that Bhopal is predominantly urban and connectivity not an issue, NGOs may be roped in or community volunteers may be provided incentives to collect and transport the sputum to NRL as per programme guidelines [10]. iii) improving tracking of referred patients through cohort-wise analysis with recording of TB registration number, wherever possible, at all levels of DTP [26] and iv) systematic qualitative enquiry into provider and patient level perspectives of reasons for attrition/delay.

## Conclusion

This OR assessed the gaps and operational challenges in diagnostic pathway of patients with presumptive MDR-TB from eligibility for DST to diagnosis. The factors identified with pre-diagnosis attrition, especially identification/referral/sample transport at field level, need to be addressed in Bhopal. RNTCP needs to intensively monitor the DTP of presumptive MDR-TB cases while expanding laboratory capacity for rapid molecular diagnosis as India gears up for universal DST to attain the target of ending TB by 2030 in line with the recently launched Sustainable Development Goals [27].

## Additional files

Additional file 1: Dataset; Description of file: This file contains all the variables of all the study particpiants that were collected during record review. (XLS 399 kb)

Additional file 2: Codebook/Data documentation sheet; Description of file: This file contains details of the fields including values (codes) and value labels. (XLSX 16 kb)

## Abbreviations

CI: Confidence interval
DMC: Designated microscopy center
DOTS: Directly observed treatment short course
DR-TB: Drug resistant tuberculosis
DST: Drug susceptibility testing
DTC: District tuberculosis center
DTP: Diagnosis and treatment pathway
FUS+: Follow up smear positive
IQR: Interquartile range
LPA: Line probe assay
MDR-TB: Multidrug resistant tuberculosis
NRL: National reference laboratory
OR: Operational research
PMDT: Programmatic management of drug resistant tuberculosis
RNTCP: Revised national tuberculosis programme
RR: Relative risk
TAT: Turnaround time
TU: Tuberculosis unit
