# Erratum to: High pre-diagnosis attrition among patients with presumptive MDR-TB: an operational research from Bhopal district, India

**DOI:** 10.1186/s12913-017-2252-x

**Published:** 2017-04-24

**Authors:** Hemant Deepak Shewade, Arun M. Kokane, Akash Ranjan Singh, Manoj Verma, Malik Parmar, Ashish Chauhan, Sanjay Singh Chahar, Manoj Tiwari, Sheeba Naz Khan, Vivek Gupta, Jaya Prasad Tripathy, Mukesh Nagar, Sanjai Kumar Singh, Pradeep Kumar Mehra, Ajay M. V. Kumar

**Affiliations:** 1 International Union Against Tuberculosis and Lung Disease (The Union), South-East Asia Office, New Delhi, India; 2 Department of Community Medicine and Family Medicine, All India Institute of Medical Sciences (AIIMS), Bhopal, India; 3 State TB cell, Department of Health and Family Welfare, Bhopal, India; 4 World Health Organization, Country Office in India, New Delhi, India; 5 Department of Community Ophthalmology, All India Institute of Medical Sciences (AIIMS), New Delhi, India; 6 International Union Against Tuberculosis and Lung Disease, Paris, France

## Erratum

Upon publication of the original article [1], it was noticed that the author’s name “Hemant Deepak Shewade” was incorrectly given as “Deepak Shewade”. This has now been acknowledged and corrected in this erratum.
